# Direct measurement of nonlocal entanglement of two-qubit spin quantum states

**DOI:** 10.1038/srep19482

**Published:** 2016-01-18

**Authors:** Liu-Yong Cheng, Guo-Hui Yang, Qi Guo, Hong-Fu Wang, Shou Zhang

**Affiliations:** 1 School of Physics and Information Engineering, Shanxi Normal University, Linfen, Shanxi 041004, China; 2 Department of Physics, Harbin Institute of Technology, Harbin, Heilongjiang 150001, China; 3 Department of Physics, College of Science, Yanbian University, Yanji, Jilin 133002, China

## Abstract

We propose efficient schemes of direct concurrence measurement for two-qubit spin and photon-polarization entangled states via the interaction between single-photon pulses and nitrogen-vacancy (NV) centers in diamond embedded in optical microcavities. For different entangled-state types, diversified quantum devices and operations are designed accordingly. The initial unknown entangled states are possessed by two spatially separated participants, and nonlocal spin (polarization) entanglement can be measured with the aid of detection probabilities of photon (NV center) states. This non-demolition entanglement measurement manner makes initial entangled particle-pair avoid complete annihilation but evolve into corresponding maximally entangled states. Moreover, joint inter-qubit operation or global qubit readout is not required for the presented schemes and the final analyses inform favorable performance under the current parameters conditions in laboratory. The unique advantages of spin qubits assure our schemes wide potential applications in spin-based solid quantum information and computation.

With the development of quantum theory, entanglement has been accepted as a characteristic physical resource in microscopic quantum systems and widely applied in the current fiery quantum information and computing or testing the fundamental principles of quantum mechanics^1^. In early stage researches, we can see that the maximally entangled pure states, like Bell states, play vital roles as initially employed resources or quantum channels in a multitude of quantum information tasks^2^,^3^. However, the maximally entangled states are difficult to transmit and store due to absorption and radiation of microparticles or diversified decoherence effect in quantum noisy channel, which inevitably impair the system entanglement and may even result in mixed states from initial entangled pure states. Consequently, in subsequent studies, researchers began to consider realizing quantum information processing tasks with specific nonmaximally entangled states^4^,^5^,^6^,^7^. This means that different degrees of entanglement can have different potential applications. Thus it is necessary to detect and quantify the entanglement of various entangled states. There have been several workable entanglement measure criterions^8^. An enlightening example is the entanglement of formation (EOF) introduced by Bennett *et al*.^9^. Afterwards, Wootters *et al*. proposed an exactly calculable quantity on the basis of EOF to quantify the entanglement of an arbitrary two-qubit state *ρ*_12_, i.e., concurrence, which can be defined as *C* = 2 max{0, *λ*_1_ − *λ*_2_ − *λ*_3_ − *λ*_4_}, where *λ*_*i*_ (*i* = 1, 2, 3, 4) are the square roots of the eigenvalues of the operator 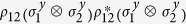 in decreasing order^10^,^11^. For an arbitrary two-qubit pure state 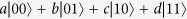, its concurrence can be worked out as 2|*ad* − *bc*|. Consequently, the concurrence of other specific two-qubit entangled states like Bell-like states can also be derived straightforwardly.

For the entangled states whose compositions are known, its entanglement can be calculated by the state parameters, regardless of which entanglement measure criterion is appointed. A complete tomographic reconstruction of the initial quantum state enable one to get the concrete parameters and figure out its degrees of entanglement^12^, but this approach requires taxing measurement of system observable especially for multiqubit entangled states. As a consequence, how to effectively quantify the entanglement of unknown quantum states becomes a valuable research issue. In recent years, a number of theoretical and experimental schemes have been presented to direct measurement of concurrence for two-qubit entangled states^13^,^14^,^15^,^16^,^17^, including pure states and mixed states. These existing researches mainly focused on measurement of entanglement for two-photon entangled states with optical elements and atomic entangled states with cavity quantum electrodynamics system^18^,^19^. Besides the local entanglement, Zhou and Sheng discussed an efficient way for measuring nonlocal entanglement of atomic two-qubit pure states^20^.

It’s generally accepted that the main properties of splendid qubits are long coherence time joining up with well manipulability. Recent years, spin qubit is supposed to be a desirable candidate for quantum information processing because of its scalability and stability. For instance, recent researches confirm that electron spin states in diamond nitrogen-vacancy (NV) center have a long lifetime even at room temperature and can be manipulated and detected by microwave or optical field^21^,^22^. These unique characteristics make the NV center one of the most potential carrier of quantum information^23^,^24^,^25^,^26^,^27^,^28^,^29^. So studying the universal and nondemolition methods of entanglement measurement for spin-qubit system will be of great value, especially under the case that only few works refers to it^30^. In this paper, we present several schemes of directly measuring concurrence for two-qubit nonlocal entangled states, including two-qubit pure states and mixed states. First, we implement non-destructive entanglement measurement for NV centers spin entangled-state with the aid of interaction between single-photon pulse and NV center embedded in microtoroidal resonator (MTR). And two spatially separated participants receive nonlocal multi-qubit maximally entangled states after the concurrence measurement protocols. In this sense, our schemes can also be considered as processes of extracting the maximally entangled states from initial partially entangled states with certain probabilities. Furthermore, we consider the direct measurement of nonlocal entanglement for two-photon polarization entangled-state via photon-NV center interactions in turn. And the proposed schemes just involve ordinary single-qubit operations and measurement without any complicated two-qubit or multi-qubit quantum logical gate. Moreover, strong coupling strength or high-quality resonator is not requisite for physical setups considered here and the long coherence time of NV center electron state at higher temperature might relieve the cryogenic requirements, which greatly reduce the experimental difficulty. Finally, we analyze the influence of imprecise operating process and less-than-ideal parameters on our schemes.

## Results

### The rationale for basic physical system

**Figure 1:**
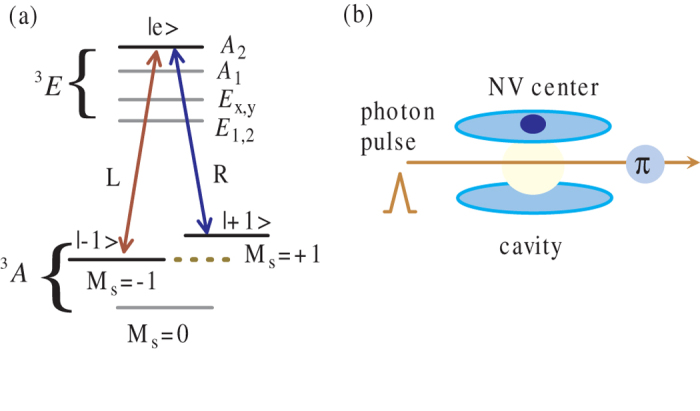
(**a**) The electron energy level configuration of the NV center and relevant transition coupling with corresponding photon polarization. 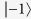 and 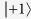 are used as qubits. (**b**) Basic model containing an NV center confined to a resonator and a single photon pulse introduced to interact with the NV center.

To begin with, we will provide a brief background on the basic unit of the NV center and microcavity system considered here. As illustrated in Fig. 1, an NV center, consisting of a substitutional nitrogen atom and an adjacent vacancy, is fixed on the surface of a microtoroidal resonator and can be coupled to the cavity mode. The electronic spin states of NV center are shown in Fig. 1(a) with the spin triplet ^3^*A* ground states and spin triplet ^3^*E* excited state. The zero-field splitting and the Zeeman splitting from an external magnetic field determine the ground levels 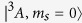, 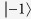 and 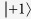
24,31. We encode the information on the ground states 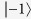 and 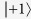 and select the excited state 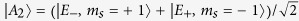 as auxiliary level 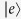. According to the decay properties of 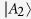, The level transition between 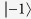 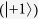 and 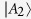 is resonantly coupled to the left (right) circularly polarized cavity mode 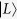 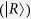, which provides a Λ-type three-level system.

Considering a single photon pulse with frequency *ω*_*p*_ to input the MTR, under the assumptions of weak excitation limit and zero vacuum input field, the reflection coefficient for the incident photon after its interactions with NV center can be described as^26^,^32^


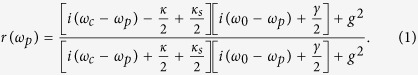


where *ω*_0_ and *ω*_*C*_ are the frequencies of the electronic energy levels transition and the cavity field; *g* is the photon-NV coupling strength; *κ, κ*_*s*_ and *γ* are the cavity decay rate, leak rate and the NV center dipole decay rate.

If the input pulse encounters an NV center in the detuning level, i.e. *g* = 0, Eq. (1) changes to


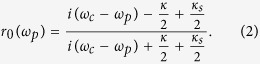


From the transition paths in Fig 1(a), if a single polarized photon 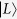 is reflected from a cavity with an NV center prepared in 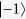 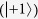, the output photon will experience a phase shift *e*^*iθ*^ 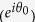 owing to optical Faraday rotation, where *θ* and *θ*_0_ indicate the arguments of complex numbers *r*(*ω*_*p*_) and *r*_0_(*ω*_*p*_). While a single polarized photon 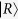 will experience a phase shift 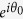 regardless of the electronic spin states, because the spin states are decoupled to input pulse due to the detuning or polarization mismatch. Parameterizing *ω*_0_ = *ω*_*C*_ = *ω*_*p*_, *κ*_*s*_ ≪ *κ* and 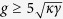, we approximately have *θ* = 0 and *θ*_0_ = *π* from Eq. (1). And here it can be seen that the strong coupling condition *g* ≫ (*κ*,*γ*) is not required. On that basis, a *π* phase shifter on the photon reflection path leads to a controlled phase flip gate on the incident photon pulse and NV center as


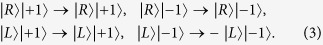


The schematic setup refers to Fig. 1(b).

### Direct measurement of concurrence with NV-microresonator system

**Figure 2:**
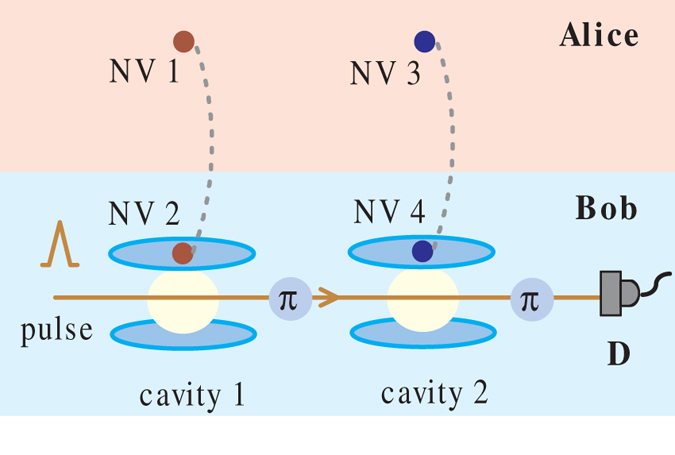
Schematic of direct measurement setups for two-NV center spin Bell-like state. The dotted lines indicate the initially entangled relationship. D: photon detector.

Based on the above NV-MTR interaction system, from now on, we elaborate the direct measurement of nonlocal entanglement of two-qubit entangled states, including pure states and mixed states. This is significant to the actually prepared microcosmic physical systems. For instance, the initial maximally entangled pure states might decay into the partially entangled pure states or even mixed states by reason of the channel noise effects in the entanglement distribution process between spatially separated participants. And we discuss now is how to obtain the concurrence of these damped unknown entangled states. First, we consider the simplest Bell-like states and assume two pairs of NV centers (1, 2) and (3, 4) in the states


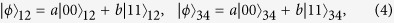


where coefficients satisfy |*a*|^2^ + |*b*|^2^ = 1, and we define 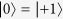 and 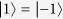 for convenience. As shown in Fig. 2, NV centers (1, 3) and (2, 4) are possessed by two spatially separate parties Alice and Bob, respectively. To begin with, Bob introduces a single photon pulse with the initial state 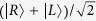 and guides the photon to interact with NV centers 2 and 4 in cavities 1 and 2 in sequence. On the basis of the controlled phase flip effect in Eq. (3), the whole system of four NV centers and an incident photon evolves to


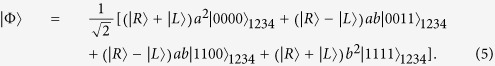


And then Bob detects the polarization state of the output photon with a single-photon detector. The obvious inference is that once the detector receives the state 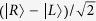, the four NV centers in separated participants collapse to a 4-qubit Greenberger-Horne-Zeilinger(GHZ) state


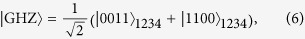


with probability


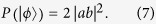


Combine the concurrence formula so we can get the relationship


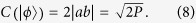


That is, the concurrence of initial spin-entangled states Eq. (4) can be obtained by the relevant detection probability of the single-photon state. It is also worth noting that this operating procedure in Fig. 2 not only presents an entanglement measurement manner, but also gives a derivative, nonlocal 4-qubit GHZ state with a success probability of Eq. (7). This is equivalent to building a 4-qubit maximally entangled channel between two participants over a long distance, which makes it possible to implement other subsequent remote quantum information processing tasks.

Besides the Bell-like state in Eq. (4), the declarative concurrence measurement scheme also applies to the other three ones. Taking 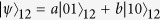 as an example, we can get the evolution result after Bob’s photon-NV centers interaction in Fig. 2 as


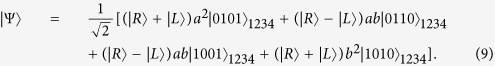


Thus the terminal detector determines the measurement result 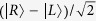 with a probability 
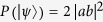
 and leaves a nonlocal 4-qubit GHZ state 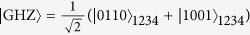. Similarly, the concurrence of 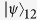 can be obtained as 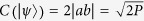.

For the two-qubit Bell-like states, the procedure of direct measurement of concurrence is relatively plain. The next unescapable consideration should be the arbitrary two-qubit entangled pure states


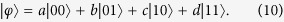


**Figure 3:**
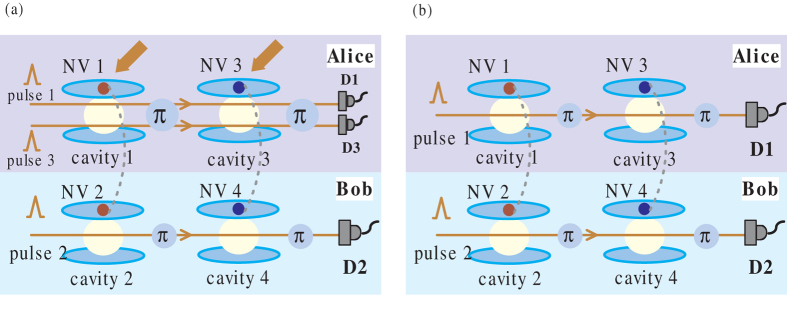
(**a**) Schematic of direct measurement scheme of concurrence for arbitrary two-qubit spin entangled pure state. (**b**) Schematic of direct measurement scheme for two-qubit spin mixed state, Collins-Gisin state.

Assume that there are two pairs of NV center spin-entangled states in Eq. (10), 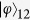 and 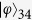, possessed by Alice and Bob. We notice that only one photon pulse adopted by Bob is not enough to measure the entanglement, but Both participants need to perform appropriate operations. The detailed process is shown in Fig. 3(a). First, Alice and Bob introduce respectively the single-photon pusles 1 and 2, which interact with NV centers (1, 3) and (2, 4) in their own spatial pathes. After the interaction process in Eq. (3), these two photons are detected by D1 and D2. If both the results of two detectors appear as 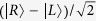, the state of NV centers becomes


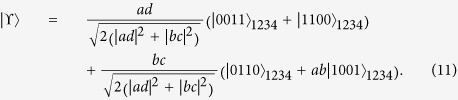


Next, Alice performs two Hadamard operators on her NV centers 1 and 3 with microwave pulses and then introduces the third single-photon pulse to pass through cavities 1 and 3. After the interaction with NV centers 1 and 3, the photon 3 is measured by D3. If the measurement result remains 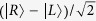, the state of four NV centers evolves into


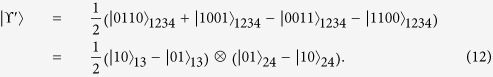


It can be calculated that the total probability of getting the state (12) is


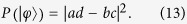


On the basis of the concurrence formula of the arbitrary two-qubit pure states, we would get the entanglement of the initial state (10) as


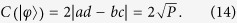


In addition, this process not only obtains the concurrence of initial state, but also gives secondary products, two local Bell states 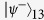 and 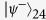.

As mentioned earlier, the environmental noises can even alter an initially pure entangled state into a mixed state sometimes. For example, the Bell-like state in Eq. (4) may evolve into the mixed state 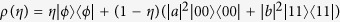 in a bit-flipping noise environment. So to measure the degree of entanglement of the two-qubit mixed state is also a ponderable question of being. For simplicity, we take a special quantum mixed states called Collins-Gisin state,


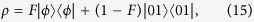


as an example and design the complete operating process to directly measure its concurrence as shown in Fig. 3(b). And it can be proved that the concurrence of other two-qubit mixed state like *ρ*(*η*) can also be measured in a similar way. Concretely speaking, consider two identical Collins-Gisin states *ρ*_1_ and *ρ*_2_ shared by Alice and Bob. The concurrence of *ρ* is directly 2*F*|*ab*|. Then the joint state of four NV centers can be expressed as


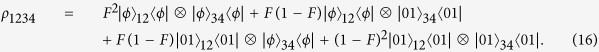


In Fig. 3(b), our scheme focuses on single-photon pulses 1 and 2 introduced by Alice and Bob respectively. The system interaction is similar to the first step in Fig. 3(a). After the output of pulses from cavities 3 and 4, the four components of Eq. (16) with input pulses evolve as


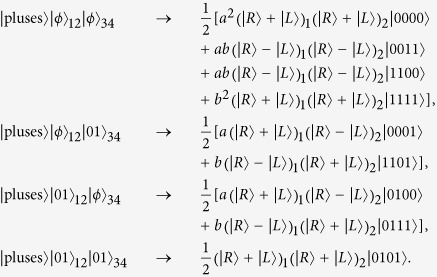


So the coincidence measurement results 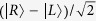 of the endmost detectors collapse state (16) as a nonlocal 4-qubit GHZ state


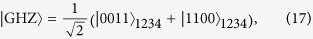


with a probability


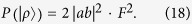


And we can get the concurrence as 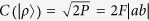 accordingly.

**Figure 4:**
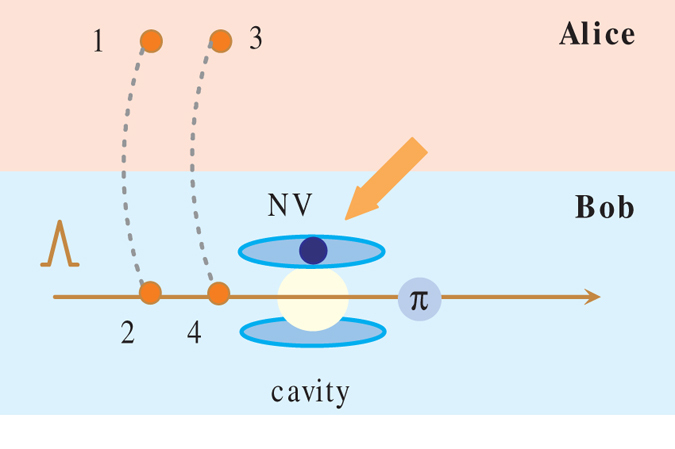
Schematic of direct measurement setups for two-photon polarization Bell-like state.

In the schemes above, we use the photon pulse as auxiliary to directly measure the concurrence of NV centers spin-entangled states. Interestingly, we find that the concurrence of two-photon polarization-entangled states can also be measured assisted by electron spin of an NV center confined on the MTR surface. The schematic and device description are shown in Fig. 4. We assume two identical photon pairs (1, 2) and (3, 4) held by Alice and Bob are initially prepared in


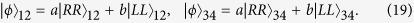


Bob first establishes an NV-MTR system with the NV center in 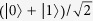. And then photons 2 and 4 pass the MTR and interact with NV center in tandem. The whole system of four photons and the NV center evolves to


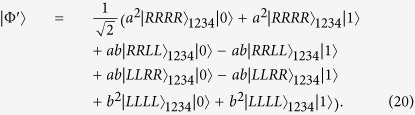


Then Bob needs to apply a Hadamard gate on the electron spin state of NV center by a *π*/2 microwave pulse, which expresses as 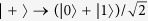 and 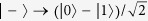. As a result, the joint state in Eq. (20) becomes


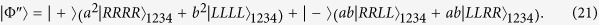


This means that the probability to detect the spin state of NV center in 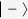 is also 
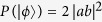
. Thus the concurrence of the initial photon polarization states can be expressed as 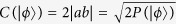. The spin readout measurement can be achieved via resonant optical excitation as discussed in refs 21,22,24. What need add is, one can also directly measure the concurrence of two-photon arbitrary entangled pure states and two-photon mixed entangled states with similar operations in Fig. 3 and NV centers as auxiliaries. And it is necessary to note here that we need to watch out for the time interval *t* between photons sequentially entering the MTR. Because if the practical operations can not meet the condition that *t* is much shorter than the electron spin relaxation time *τ*, it will impact the efficient photon-NV center interaction and give imprecise results of Eq. (3). Fortunately, it has been confirmed that the electron spin relaxation time and dephasing time of NV centers in diamond are relatively long (ms) even at room temperature, which might ensure enough operating time^22^,^33^,^34^.

So far, we have discussed several schemes of direct measurement concurrence of two-qubit entangled states, including nonlocal NV center spin states and photon polarization states. As can be seen from Figs 2, 3, 4, we design different operations to different initial entangled-states. It is necessary to note that the measurement scheme in Fig. 3(a) is appropriate for the arbitrary two-qubit entangled pure states, thus it also suits the Bell-like states and can be proved to be applicable to the mixed state in Eq. (15) as well. This makes the scheme of Fig. 3(a) be an universal method to directly measure the concurrence of initial two-qubit states. And in the context of knowing entangled type (without the need for parameters), we can choose an appropriate measurement scheme relatively simple.

## Discussion

**Figure 5:**
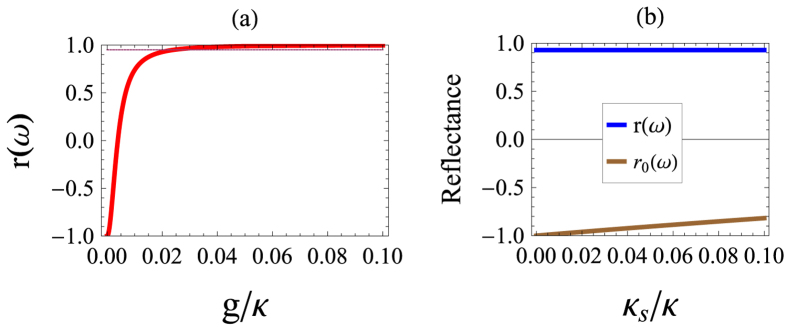
The reflection coefficient as functions of accessible system parameters under the resonant condition *ω*_0_ = *ω*_*C*_ = *ω*_*p*_. (**a**) *r*(*ω*_*p*_) against *g*/*κ* for *γ* = 1.5 × 10^−5^*κ, κ*_*s*_ ≈ 0. The dotted lines indicates *r*(*ω*_*p*_) = 0.95. (**b**) *r*(*ω*_*p*_) against *κ*_*s*_/*κ* for *γ* = 1.5 × 10^−5^*κ* and *g* = 0.01*κ*.

In the presented schemes, the precise entanglement measurement depends on the interaction results in Eq. (3). That is, the reflectance of output photon pulse requires *r*(*ω*_*p*_) = *e*^*iθ*^ = 1 and 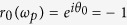, i.e., *θ* = 0 and *θ*_0_ = *π*. In consequence, it is worthwhile to consider the effect of imprecise coupling strength and decay factors on our schemes in practically available physical system. In Fig. 5(a), we first verify the reflectance against the ratio of coupling strength to cavity decay rate taking *κ*_*s*_ ≪ *κ, γ* = 1.5 × 10^−5^*κ*. It shows that the reflectance *r*(*ω*_*p*_) ≈ 0.95 even when *g* ≈ 0.01*κ*. This means the presented schemes do not essentially need strong coupling. And the involved parameter values are available in current solid-state microcavity experiment^35^. Figure 5(b) shows the influence of side leak rate on the reflectance. The coupled and decoupled reflectances are valid when *κ*_*s*_ is negligible compared with the main cavity decay *κ*, e.g., *κ*_*s*_ = 0.02*κ* leads to *r*(*ω*_*p*_) ≈ 0.93 and *r*_0_(*ω*_*p*_) ≈ −0.96.

Figure 5 shows that the imperfect coupling strength and leak rate will inevitably bring about slight influence on the reflectance of output photons. So the interaction of the incident photon pulse and NV center in Eq. (3) should be rewritten as


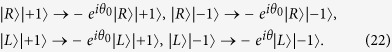


Here, taking the first measurement scheme in Fig. 2 as an example, we calculate the success probability of getting the detection result 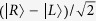 as


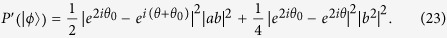


**Figure 6:**
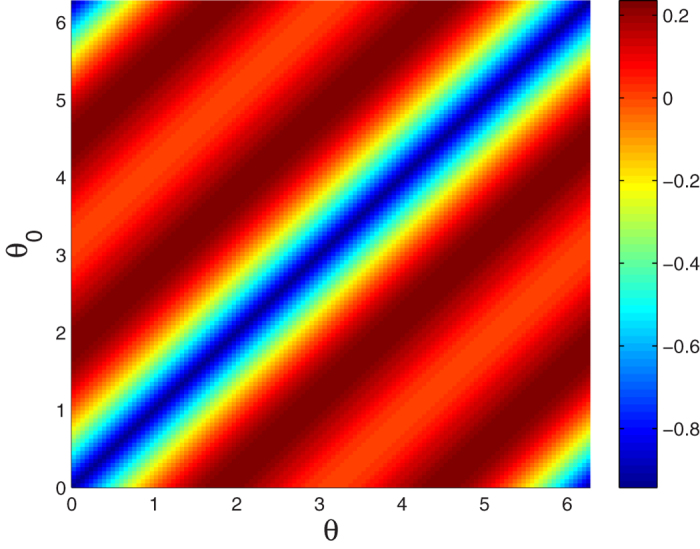
The concurrence deviation between the results under conditions (*θ* = 0, *θ*_0_ = *π*) and imperfect conditions as a function of *θ* and *θ*_0_ for 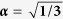.

In order to see clearly the effect of imprecise reflection coefficients on our scheme, we plot the variation trend of concurrence deviation 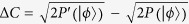 between the calculation results in ideal conditions (*θ* = 0, *θ*_0_ = *π*) and imperfect conditions against *θ* and *θ*_0_ in Fig. 6. It is observed that if the difference of *θ* and *θ*_0_ is close to 0 or 2*π*, 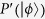 will approximate to 0 and the absolute value of Δ*C* equals to 
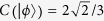
. But if the difference of *θ* and *θ*_0_ is close to *π* (approximate to ideal value), Δ*C* will tend to be 0. It means that the concurrence actually obtained equals to the original value. As an example, the experimental parameters under current technical condition can achieve *r*(*ω*_*p*_) ≈ 0.93 and *r*_0_(*ω*_*p*_) ≈ −0.96, which render a slight deviation Δ*C* < 0.09.

**Figure 7:**
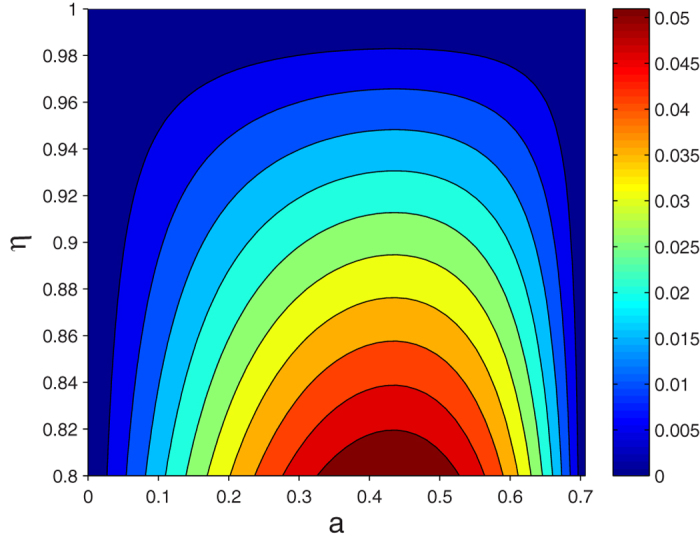
The concurrence deviation between the results under identical initial states conditions and non-identical conditions as a function of coefficient *a* and the scale factor *η*.

One other thing to note here is that our presented concurrence measurement schemes require two pairs of identical initial entangled states, on which appropriate operations and detections are performed to measure entanglement. However, during practical preparation process, it is possible to obtain two similar but not identical initial states even with the same physical devices and operations. Next, we will consider the influence of the initial coefficients deviation on the concurrence measurement. Assume that for the first scheme, NV centers (1, 2) and (3, 4) shared by Alice and Bob are prepared in


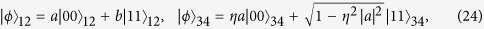


where *η* is a scale factor. After the operation process in Fig. 2, we can get a probability of receiving the detecting result 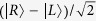 as


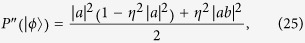


and then work out the concurrence in this case as 
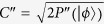
. Compared with the calculation results in identical initial states, we plot the variation trend of concurrence deviation 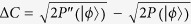 against the coefficient *a* and the scale factor *η* in Fig. 7. It is observed that there is little difference between the actually obtained concurrence and the ideal one for *η* ∈ (0.8, 1). Using the analogous method, we can also verify the influence of the initial coefficients deviation on the concurrence measurement of the mixed Collins-Gisin states. Put simply, the actually obtained concurrence increases with the increase of the coefficient *F* in Eq. (15). It would be easy enough to comprehend that the calculated concurrence depends on the probability of detection results 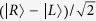 for two detectors, and the conclusive components of this detection result exist in the first term of Collins-Gisin state. Therefore, increasing the proportion of in the first term leads to the bigger detection probability and calculated concurrence, and vice versa.

For actual experiment technologies, the inefficiency of detection process should be considered, which will bring ineffectiveness to our schemes. In realistic operations, one can continuously inject photon to the setups until the photon detector is triggered within the time scale of the electron-spin coherence time. It is confirmed that the electron spin relaxation time and the dephasing time of NV centers can be milliseconds at room temperature in recent researches^22^,^33^,^34^. On the other hand, recent experimental technology can generate 300000 high quality single photons within 30 seconds^36^, which ensures our schemes can be accomplished within the valid duration. Moreover, the current equipment technology of optical resonator can make the mode leak rate *κ*_*s*_ negligible compared to the decay rate in output mode *κ*.

In summary, we have proposed several schemes of directly measuring concurrence for two-qubit spin and polarization entangled states using the physics system of photon-NV center interaction. The discussed entangled states include two NV centers spin Bell-like states, two-photon polarization Bell-like states, arbitrary two-qubit spin entangled pure states and two-qubit spin mixed states. These schemes can directly measure the nonlocal entanglement owned by two spatially separated participants by virtue of auxiliary single-photon pulse or NV center. The original systems are not completely destroyed by the measurement schemes, but collapsed to relevant multi-qubit maximally entangled states, which can be used as efficient entangled resources for subsequent quantum information processing tasks. In this sense, our schemes can also be considered as processes of extracting the maximally entangled states from initial partially entangled states with certain probabilities. And the proposed schemes just involve ordinary single-qubit operations and measurement without any complicated two-qubit or multi-qubit quantum logical gate. Moreover, strong coupling strength or high-quality resonator is not requisite for physical setups considered here and the long coherence time of NV center electron state at higher temperature might relieve the cryogenic requirements, which greatly reduce the experimental difficulty. Finally, we analyzed the influence of imprecise operating process and parameters on the presented schemes. The analyses showed that our schemes are feasible under the current experimental conditions.

## Additional Information

**How to cite this article**: Cheng, L.-Y. *et al*. Direct measurement of nonlocal entanglement of two-qubit spin quantum states. *Sci. Rep.* **6**, 19482; doi: 10.1038/srep19482 (2016).
